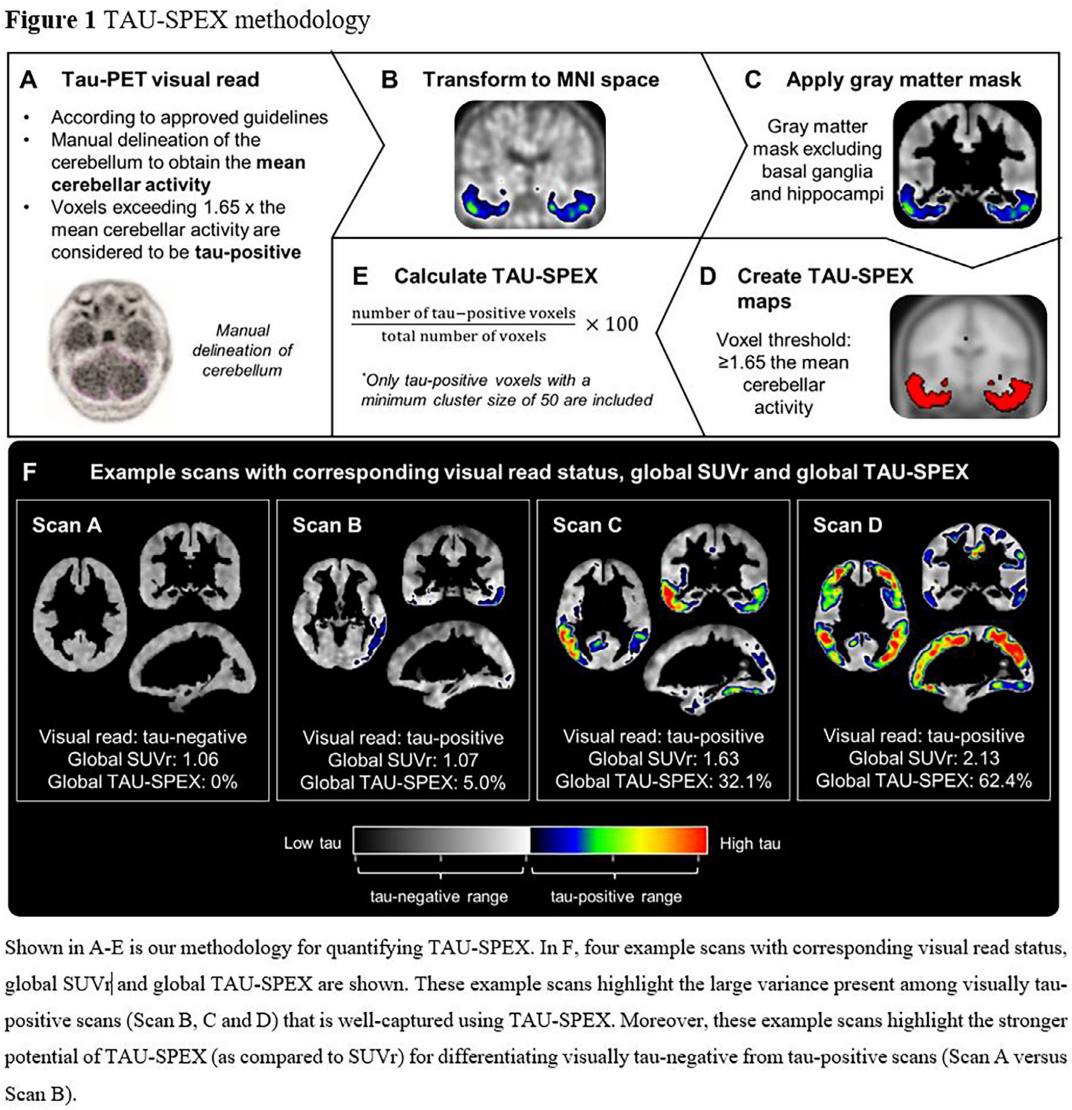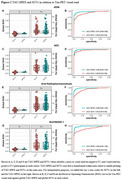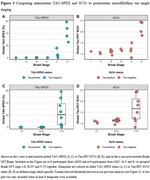# Quantitation of PET spatial extent as a potential adjunct to visual interpretation of [^18^F]flortaucipir imaging

**DOI:** 10.1002/alz70856_106609

**Published:** 2026-01-07

**Authors:** Emma M. Coomans, Bastaan van Tol, Colin Groot, Ruben Smith, Sebastian Palmqvist, Erik Stomrud, Michael Pontecorvo, Sergey Shcherbinin, Ian A. Kennedy, Vikas Kotari, Wiesje M. van der Flier, Yolande A.L. Pijnenburg, Niklas Mattsson‐Carlgren, Oskar Hansson, Elsmarieke van de Giessen, Rik Ossenkoppele

**Affiliations:** ^1^ Amsterdam Neuroscience, Neurodegeneration, Amsterdam, Netherlands; ^2^ Department of Neurology, Alzheimer Center Amsterdam, Amsterdam Neuroscience, Vrije Universiteit Amsterdam, Amsterdam, Netherlands; ^3^ Alzheimer Center Amsterdam, Neurology, Vrije Universiteit Amsterdam, Amsterdam UMC location VUmc, Amsterdam, Netherlands; ^4^ Clinical Memory Research Unit, Lund University, Malmö, Skåne, Sweden; ^5^ Memory Clinic, Skåne University Hospital, Malmö, Skåne, Sweden; ^6^ Clinical Memory Research Unit, Department of Clinical Sciences Malmö, Faculty of Medicine, Lund University, Lund, Sweden; ^7^ Eli Lilly and Company, Indianapolis, IN, USA; ^8^ Alzheimer Center Amsterdam, Department of Neurology, Amsterdam UMC, location VUmc, Amsterdam, Netherlands; ^9^ Wallenberg Center for Molecular Medicine, Lund University, Lund, Sweden; ^10^ Department of Radiology and Nuclear Medicine, Amsterdam UMC, Vrije Universiteit Amsterdam, Amsterdam Neuroscience, Amsterdam, Netherlands

## Abstract

**Background:**

The US Food and Drug Administration and the European Medicines Agency recently approved a visual read method for the Tau‐PET radiotracer [^18^F]flortaucipir to support the clinical diagnosis of Alzheimer's disease. However, among visually Tau‐PET‐positive scans large variation exists in the number of voxels with elevated Tau‐PET signal. Here, we propose a metric quantifying the spatial extent of Tau‐PET‐positivity (hereafter referred to as “TAU‐SPEX”) and evaluate its potential to be used as an adjunct to Tau‐PET visual read (VR) in clinic.

**Method:**

[^18^F]flortaucipir data from 1,635 participants (aged 71.9±8.2 years, 49.7% females, 42.1% cognitively unimpaired, 25.2% mild cognitively impaired, and 32.7% dementia) from four cohorts were visually read following approved guidelines. We calculated TAU‐SPEX as the percentage of gray matter voxels exceeding the tau‐positive threshold (Figure 1). The tau‐positive threshold was set at >65% the cerebellar average, similarly to the threshold used for visual reading of [^18^F]flortaucipir PET. Since Tau‐PET is typically quantified using SUVr metrics, we additionally calculated SUVr in a global region‐of‐interest. We compared TAU‐SPEX to SUVr in their concordance with VR tau‐status, relationship with tau neurofibrillary tangle (NFT) pathology in advanced Braak stages at autopsy (i.e. Braak V‐VI), and correlation with concurrent and longitudinal cognitive performance.

**Result:**

Among visually tau‐positive participants, TAU‐SPEX ranged from 0.0‐72.8% (median: 9.9%, interquartile range: 20.9%), highlighting the large variance in the extent of tau among scans with a positive Tau‐PET VR. TAU‐SPEX outperformed SUVr in differentiating VR tau‐status in all cohorts (*p* <0.05) (Figure 2). Cohen's kappa analyses showed stronger concordance for VR with TAU‐SPEX (κ=0.81‐0.90 across cohorts) than with SUVr (κ=0.64‐0.82 across cohorts). In a subset with autopsy data, TAU‐SPEX corresponded better with the presence of NFT pathology in Braak stages V‐VI (sensitivity: 87.5%; specificity: 100.0%) than SUVr (sensitivity: 62.5%; specificity: 90.0%) (Figure 3). Finally, compared to SUVr, TAU‐SPEX correlated more closely with concurrent and longitudinal cognitive functioning (ΔAIC TAU‐SPEX – SUVr = ‐43.2).

**Conclusion:**

TAU‐SPEX is an intuitive metric that could serve a dual purpose in clinic, i.e. supporting the VR for diagnostic applications and complementing the VR for prognostic purposes.